# m6A-mediated upregulation of lncRNA-AC026356.1 promotes cancer stem cell maintenance in lung adenocarcinoma via activating Wnt signaling pathway

**DOI:** 10.18632/aging.204689

**Published:** 2023-05-03

**Authors:** Zhen Zhang, Xiaoning Tan, Ruoxia Wu, Tianhao Deng, Huazhong Wang, Xiulin Jiang, Puhua Zeng, Junqi Tang

**Affiliations:** 1 Department of Oncology, Affiliated Hospital of Hunan Academy of Traditional Chinese Medicine, Changsha 410006, P.R. China; 2 School of Chinese Medicine, Hunan University of Chinese Medicine, Changsha 410208, P.R. China; 3 Department of Medicine, UF Health Cancer Center, University of Florida, Gainesville, FL 32611, USA; 4 Kunming College of Life Science, University of Chinese Academy of Sciences, Kunming, Yunnan, 650201, P.R. China; 5 Department of Respiratory Medicine, Hospital of Traditional Chinese Medicine, Affiliated to Southwest Medical University, Luzhou 646000, P.R. China

**Keywords:** lncRNA, immunotherapy, immune checkpoint inhibitors, T cell exhaustion, cancer stem cell, cisplatin resistance

## Abstract

LncRNA plays a pivotal role in the stemness and drug resistance of lung cancer. Here, we found that lncRNA-AC026356.1 was upregulated in stem spheres and chemo-resistant lung cancer cells. Our fish assay also shows that AC026356.1 was predominantly located in the cytoplasm of lung cancer cells and does not have protein-coding potential. Silencing AC026356.1 significantly inhibited proliferation and migration but increased apoptosis in A549-cisplatin (DDP) cells. Additionally, IGF2BP2 and the lncRNA-AC026356.1 positively regulated the proliferation and stemness of stem-like lung cancer cells. Further mechanistic investigation revealed that METTL14/IGF2BP2-mediated m6A modification and stabilization of the AC026356.1 RNA. Functional analysis corroborated that AC026356.1 acted as a downstream target of METTL14/IGF2BP2 and AC026356.1 silencing could block the oncogenicity of lung cancer stem-like cells. AC026356.1 expression was correlated with immune cell infiltration and T cell exhaustion. Compared with paired adjacent normal tissues, lung cancer specimens exhibited consistently upregulated METTL14/IGF2BP2/AC026356.1. M6A-modified METTL14/IGF2BP2/AC026356.1 loop may serve as a potential therapeutic target and prognostic predictor for lung cancer therapy and diagnosis in the clinic.

## INTRODUCTION

Lung cancer is a highly malignant tumor that often leads to extremely high mortality. [1, 2]. Cancer stem cells (CSCs) have been extensively studied and proved to account for multidrug resistance and metastasis in various cancer types [3]. Therefore, mechanistic studies aiming to illustrate the molecular machinery required to initiate and maintain the malignant behavior of CSCs may pave the way for the final eradication of CSCs and improve the therapeutic efficacy of lung cancer.

Long noncoding RNAs play an indispensable function in the regulate the expression of oncogenes or tumor suppressor genes [4–7]. High-throughput next-generation sequencing has revealed that N6-methyladenine is the most abundant RNA modification in diverse cells and plays a pivotal function in the gene expression [8, 9]. m6A modification exerts its function via readers (IGF2BPs, METTL3/14) and erasers (FTO, ALKBH5) [10, 11]. However, information available on the expression, regulation, and clinical significance of m6A-related AC026356.1 in LUAD has remained lacking.

In this study, we investigated how IGF2BP2 forms a positive loop with the AC026356.1 signaling cascade through modulation at transcriptional and post-transcriptional levels. On one hand, IGF2BP2 maintains AC026356.1 transcription, acting as a positive transcription factor. On the other hand, AC026356.1 promoted the transcription of METTL14/IGF2BP2 and guided m6A modification. The m6A-modified AC026356.1 loop may serve as a potential prognostic predictor for lung cancer patients.

## MATERIALS AND METHODS

### Data download

We obtained the RNA expression and clinical information of lung cancer from the TCGA-LUAD database (https://cancergenome.nih.gov/). We analysis of the expression, clinical information, and prognosis of AC026356.1 in lung cancer by using the above TCGA dataset.

### LncACTdb database

LncACTdb is an updated database of experimentally supported ceRNA interactions. In this study, we use LncACTdb to analyze the potential AC026356.1-related ceRNA network [12].

### Cell lines, cell culture, and qPCR assay

The lung cancer cell line was purchased from the cell bank of the ATCC and cultured in BEGM media (Lonza, CC-3170). The Qpcr primers as follows: AC026356.1-F: TAGAGAAAATTATTCTT; AC026356.1-R: GCTTGCCCTAACAGCAGC; AC026356.1-ASO: ATGTGAGTATAGGGCAGAT.

### Cell proliferation assay

The cholecystokinin octapeptide (CCK-8) assay was performed 24, 48, 72, and 96 h after propagation. Cells were seeded and assessed in triplicates.

### Tumor sphere formation assay

Cells (2 × 10^4^) were seeded onto 12-well plates and cultured in serum-free 1640 medium. Cell spheroids were documented and quantified using an inverted microscope (Olympus, Japan) after two weeks.

### Statistical analysis

Comparisons between groups were calculated using the Wilcoxon rank-sum or Kruskal-Wallis test. *P* < 0.05 was considered significant and marked with asterisks as indicated.

### Data availability

All data used in the study were from The Cancer Genome Atlas (TCGA-LUAD) (https://portal.gdc.cancer.gov/).

## RESULTS

### AC026356.1 is highly expressed in LUAD

**Figure 1:**
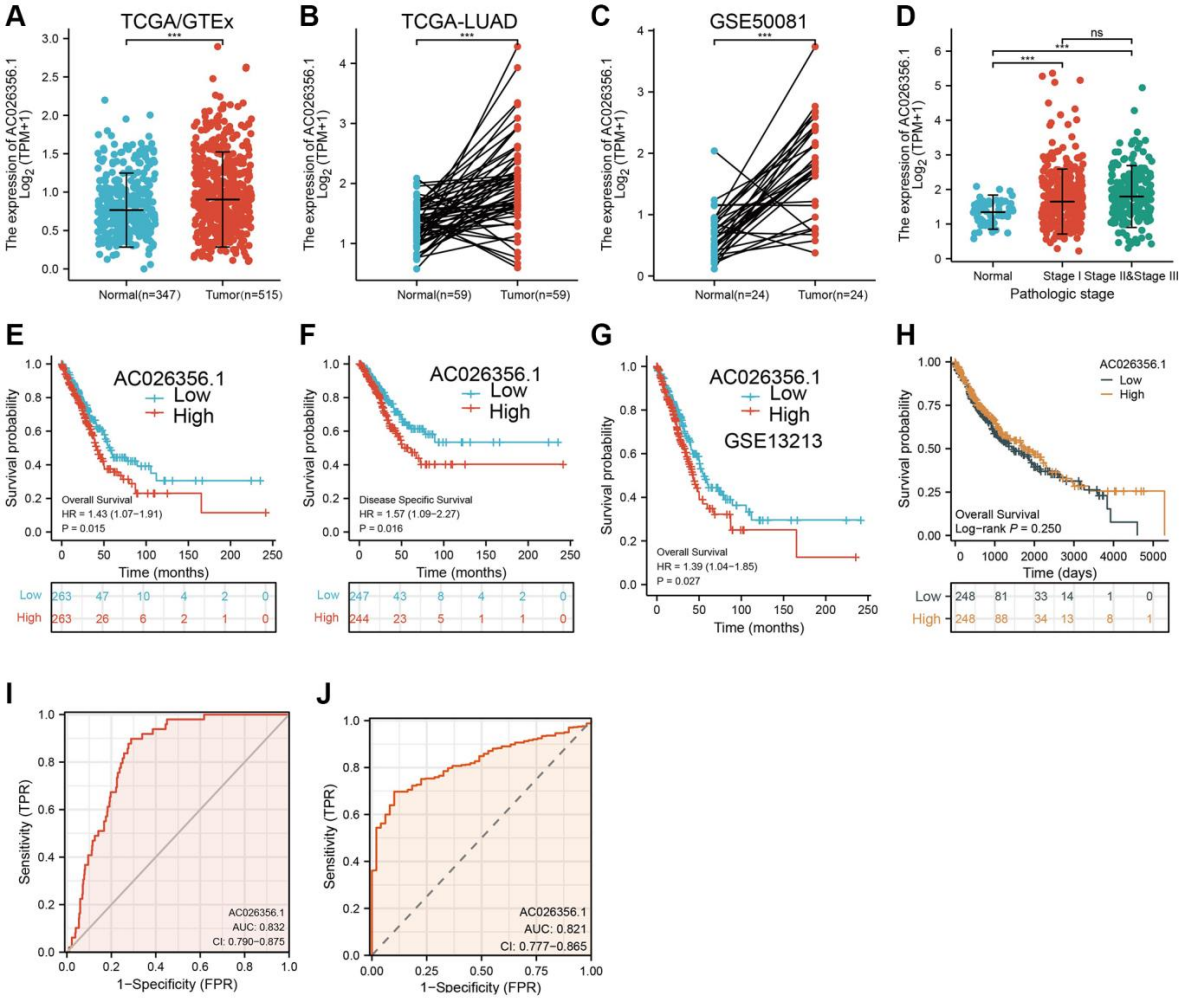
**AC026356.1 was upregulated in LUAD.** (**A**–**C**) AC026356.1 level in lung cancer by TCGA and GEO database. (**D**) Correlation between AC026356.1 expression and pathological stage in LUAD by TCGA database. (**E**–**G**) Prognosis of AC026356.1 in lung cancer examined by TCGA and GEO dataset. (**H**) Prognosis of AC026356.1 in LUSC examined by TCGA dataset. (**I**, **J**) Diagnostic value of AC026356.1 in NSCLC. NS > 0.05 and ^***^p < 0.001.

We performed data mining using the TCGA dataset and confirmed the overexpression of AC026356.1 in lung cancer samples (Figure 1A, 1B). Lung cancer transcriptome sequencing data from Gene Expression Omnibus (GEO) also showed that AC026356.1 is highly expressed in lung cancer (Figure 1C). Elevation of AC026356.1 was positively correlated with the pathological stages (Figure 1D). We next determine the relationships between AC026356.1 expression and clinical feature. Results showed that forced expression of AC026356.1 was correlated with diverse survival events (Figure 1E, 1F). These results also validate by the GEO dataset (Figure 1G). While, AC026356.1 not affect the prognosis of lung squamous cell carcinoma (LUSC) patients (Figure 1H). ROC curve analysis results uncover that AC026356.1 may be a potential diagnostic marker in lung cancer (Figure 1I, 1J).

### Analysis of subcellular localization and coding ability of AC026356.1

**Figure 2:**
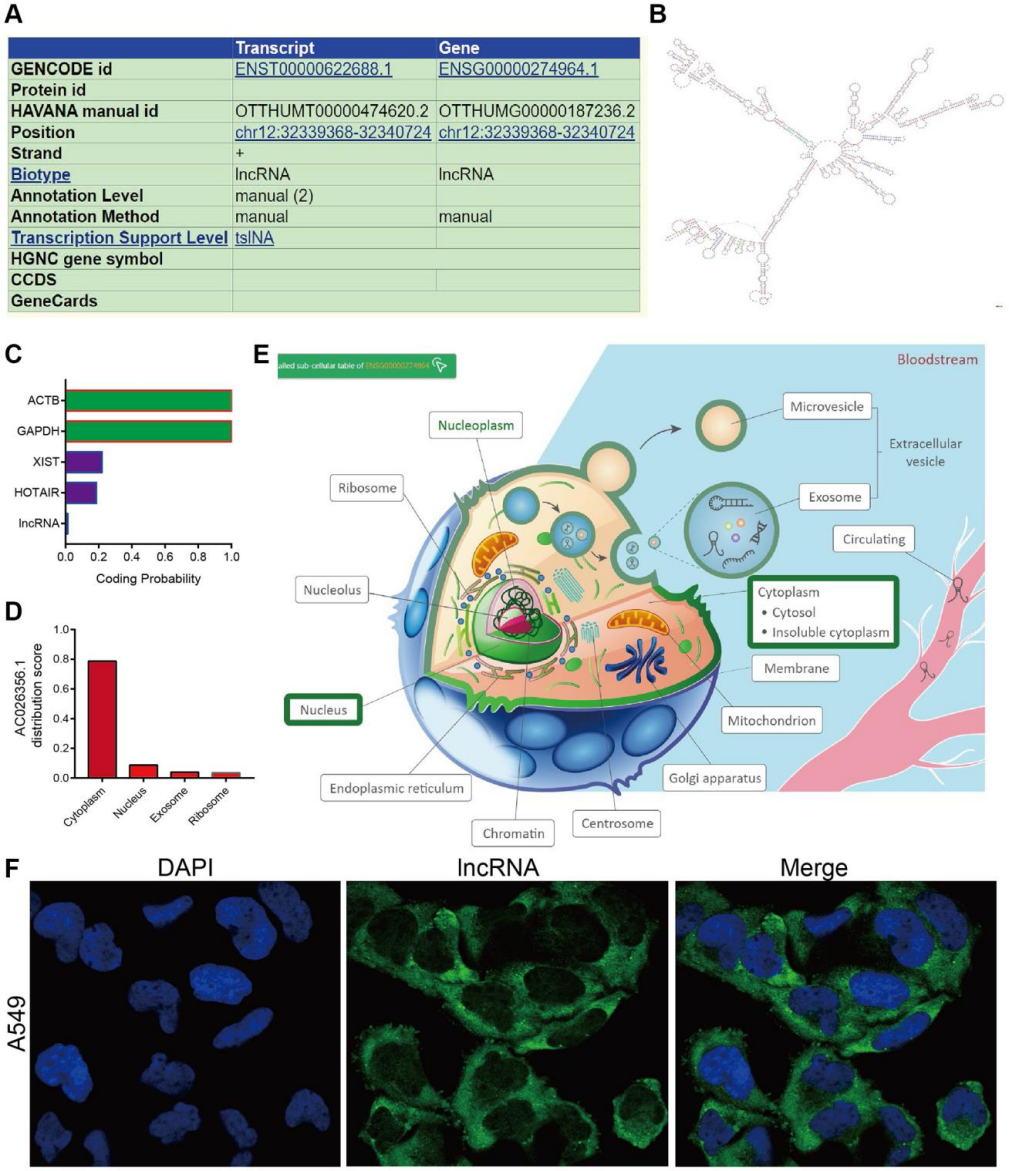
**Gene characteristics analysis of AC026356.1.** (**A**, **B**) The genetic characteristics and secondary structure of AC026356.1 by using the UCSC database (http://genome.ucsc.edu/). (**C**–**E**) Subcellular localization and coding ability analysis of AC026356.1 by the online database. (**F**) Subcellular localization of AC026356.1 by FISH assay.

We further analysis of the genetic characteristics and secondary structure of AC026356.1 by using the UCSC database (http://genome.ucsc.edu/). As is shown in Figure 2A, 2B, AC026356.1 is located in chromosome 12 and has a complex spatial structure. Subcellular localization and coding ability analysis confirmed that AC026356.1 was predominantly resided in the cytoplasm of lung cancer cells and does not have protein-coding potential (Figure 2C–2F).

### m6A modification is correlated with AC026356.1 upregulation in LUAD

**Figure 3:**
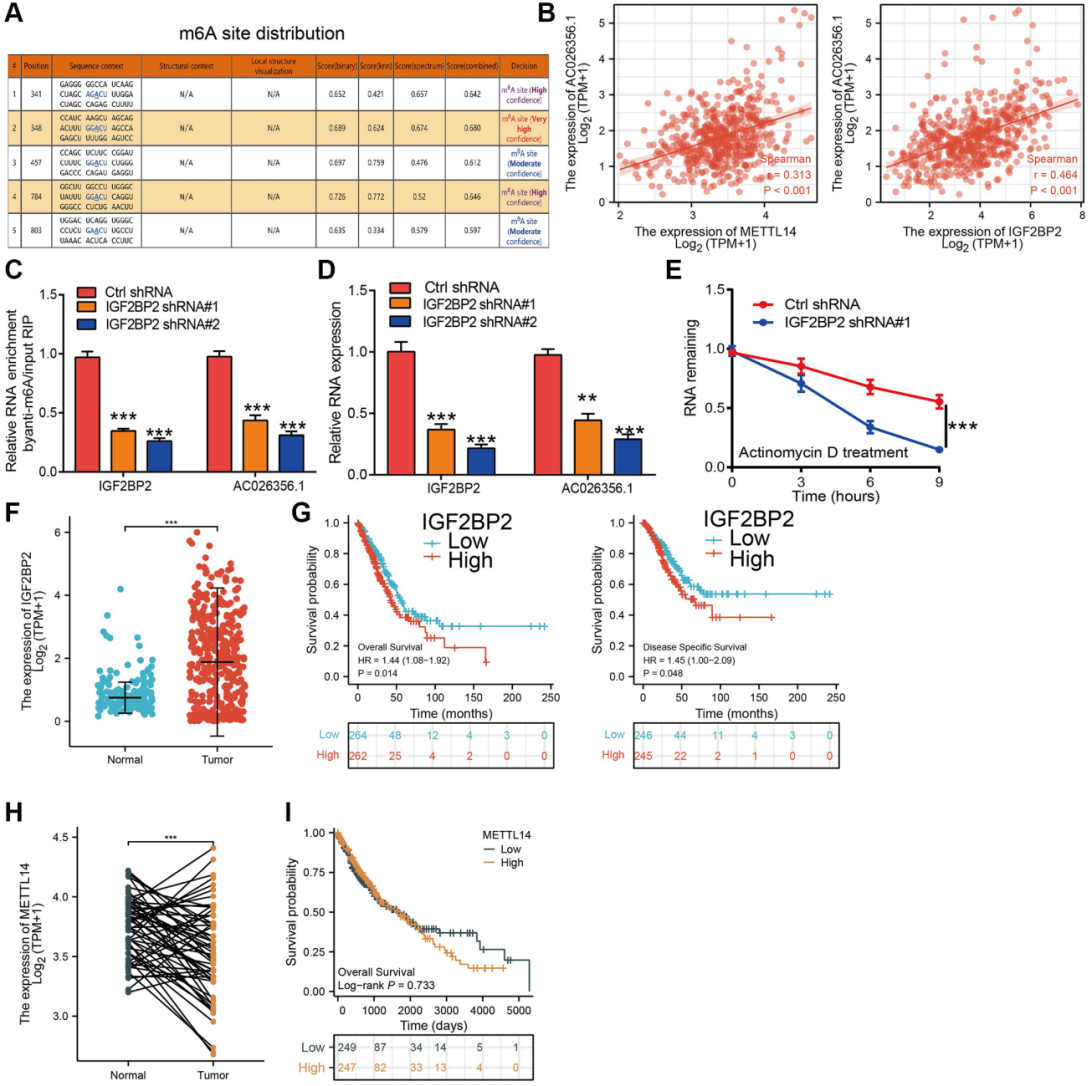
**m6A-mediated upregulation of AC026356.1 in lung adenocarcinoma.** (**A**) The RNA m6A methylation site distribution of AC026356.1. (**B**) Correlation with AC026356.1 expression and METTL14/IGF2BP2 in lung adenocarcinoma. (**C**) The m6A methylation level of AC026356.1 after depletion of IGF2BP2 in NSCLC cell lines was examined by qRT-PCR assay. (**D**) The expression of AC026356.1 after depletion of IGF2BP2 in NSCLC cell lines was examined by qRT-PCR assay. (**E**) IGF2BP2 knockdown in A549 cells significantly downregulated AC026356.1 RNA abundance. (**F**) IGF2BP2 level in lung cancer by TCGA. (**G**) Prognosis of IGF2BP2 in lung cancer examined by TCGA and GEO dataset. (**H**, **I**) Expressing and prognosis of METTL14 in lung cancer by TCGA. ^***^p < 0.001.

RNA methylation plays a core role in regulating RNA stability and translation [11]. We next explored whether m6A was associated with AC026356.1 upregulation in LUAD. Based on the results from the m6Avar database (http://www.cuilab.cn/sramp), we found diverse m6A modification sites in the exon region of AC026356.1 (Figure 3A). By analysis of TCGA-LUAD data, we show that METTL14 and IGF2BP2 were positively correlated with AC026356.1 expression in LUAD (Figure 3B). IGF2BP2 is a crucial m6A reader protein and diverse manuscript showed that IGF2BP2 could maintenance lncRNA stability [13]. To determine the effect of IGF2BP2 on AC026356.1 in LUAD, we depletion of AC026356.1 in A549 cells, we found that the m6A level of AC026356.1 was reduced in IGF2BP2-silenced LUAD cells (Figure 3C). Moreover, we uncover that IGF2BP2 knockdown was correlated with reduced AC026356.1 expression level (Figure 3D). Actinomycin D used to block transcription in lung cancer cells and uncover that knockdown of IGF2BP2 significantly reduced the half-life of AC026356.1 in A549 cells (Figure 3E). We also found that IGF2BP2 was highly expressed in lung cancer, patients with higher IGF2BP2 indicating poor prognostic in LUAD (Figure 3F, 3G). METTL14 was reduced in LUAD and not affect the prognosis of LUAD patients (Figure 3H, 3I).

### GO and KEGG analysis

**Figure 4:**
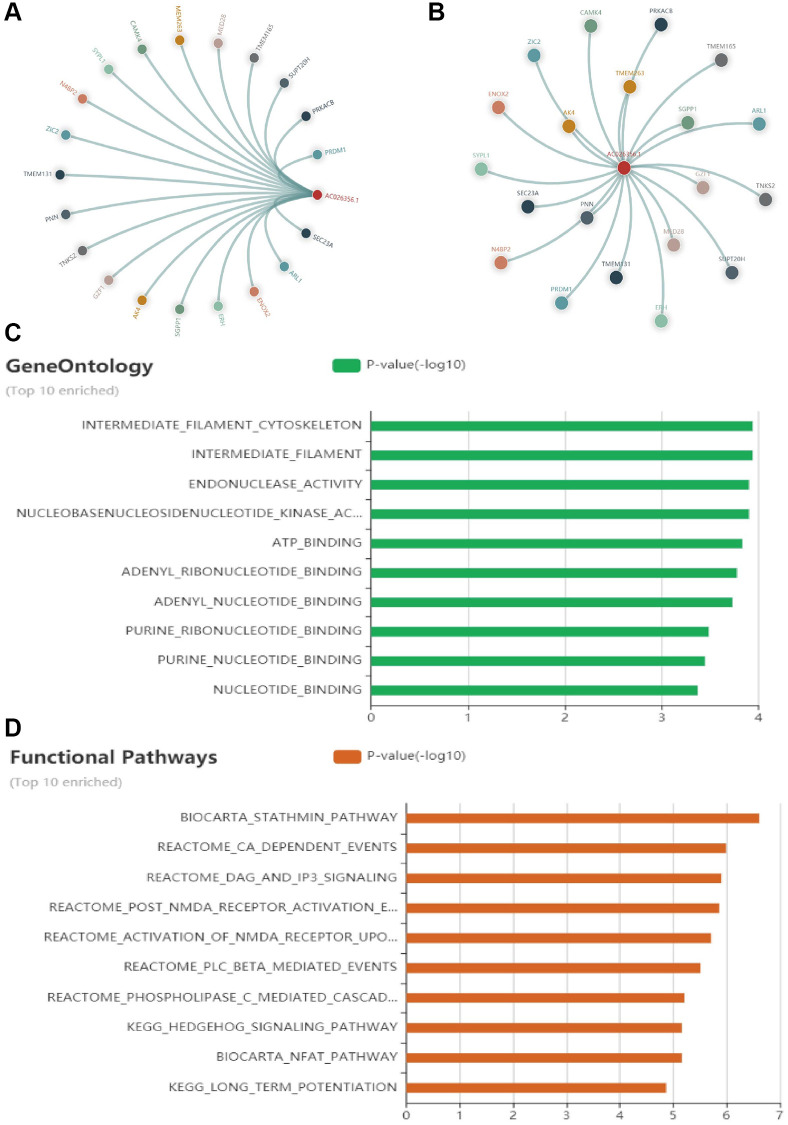
**GO and KEGG enrichment analysis.** (**A**, **B**) Positive genes with AC026356.1 in LUAD were examined by LncACTdb. (**C**, **D**) GO and KEGG enrichment analysis of AC026356.1 in LUAD examined by LncACTdb database.

To confirmed the potential function of AC026356.1 in LUAD. LncACTdb 3.0 database was used to obtain the positive genes with AC026356.1 in LUAD (Figure 4A, 4B). Moreover, we using the above genes conducted the GO and KEGG enrichment analysis. Results confirmed that AC026356.1 is major involved in the intermediate filament cytoskeleton, endonuclease activity, and nucleotide binding (Figure 4C). KEGG enrichment results showed that AC026356.1 is major involved in the hedgehog signaling (Figure 4D).

### AC026356.1 is required for malignant behaviors in LUAD cells

**Figure 5:**
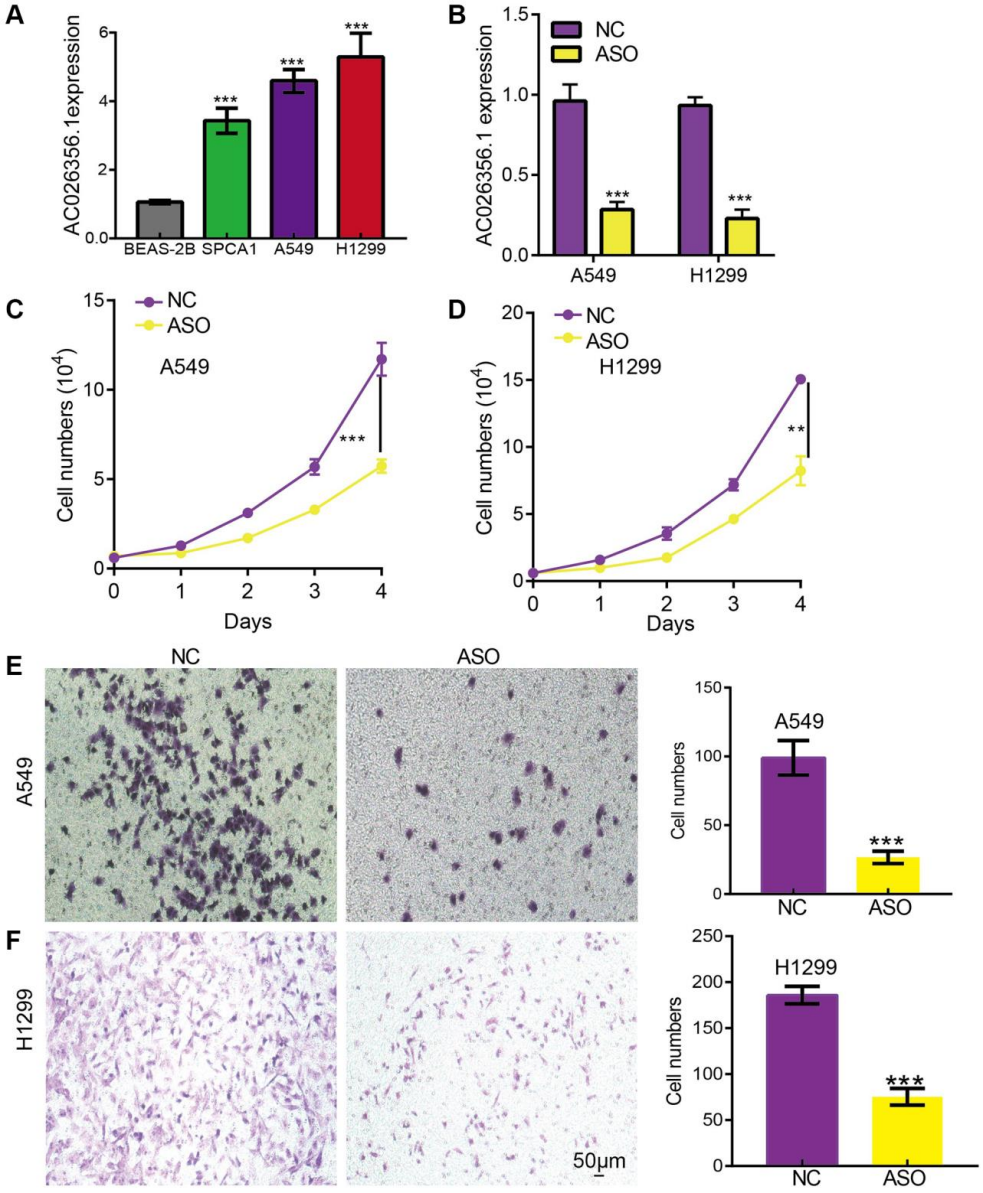
**AC026356.1 promotes LUAD progression.** (**A**) The expression of AC026356.1 in LUAD cell lines. (**B**) The knockdown efficiency was examined by qPCR assay. (**C**–**F**) Knockdown of AC026356.1 significantly inhibited LUAD cell growth and migration. ^**^p < 0.01 and ^***^p < 0.001.

We confirmed that AC026356.1 was upregulated in LUAD cell lines than bronchial epithelial cell lines (Figure 5A). ASO using to inhibited AC026356.1 expression in lung cancer cells (Figure 5B). We uncover that reduced the expression of AC026356.1 was inhibited the cell growth and migration abilities of lung cancer cells (Figure 5C–5F).

### AC026356.1 is required for the self-renewal of cancer stem cell

**Figure 6:**
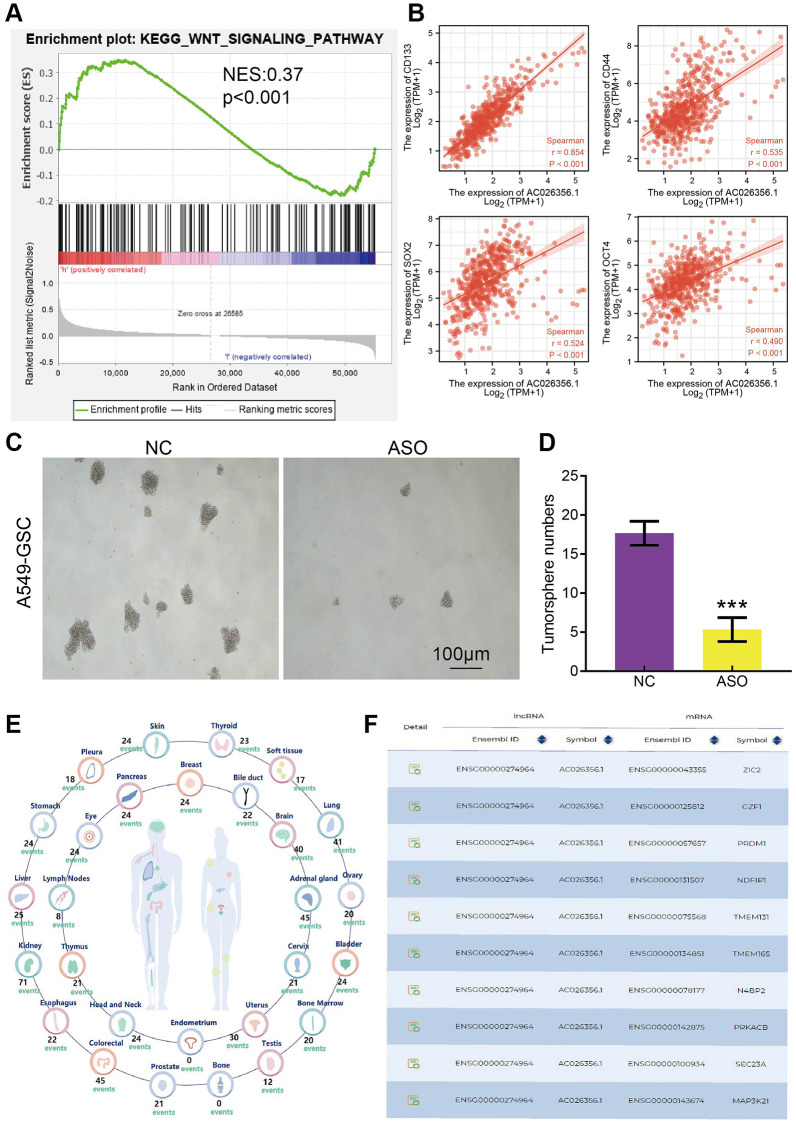
**AC026356.1 is required for the self-renewal of cancer stem cell.** (**A**) GSEA enrichment results show that AC026356.1 was involved in the Wnt signaling pathway. (**B**) Correlation between AC026356.1 and CD133, CD44, SOX2 and OCT4 in LUAD. (**C**, **D**) AC026356.1 knockdown repression significantly attenuated sphere formation of lung cancer cells. (**E**, **F**) The gene regulation by the lncRNA-miRNA-mRNA ceRNA network in the progression of LUAD. ^***^p < 0.001.

Wnt signaling pathways play a core role in maintaining the stemness of cancer stem cells [14]. Given that GSEA enrichment results show that AC026356.1 was participated in the Wnt signaling pathway (Figure 6A). By analysis of the TCGA-LUAD data, we found that AC026356.1 expression was significantly positive with the stem cell-related gene, including CD133, CD44, SOX2, and OCT4 in LUAD (Figure 6B). we further investigated how AC026356.1 functions in lung cancer stemness. AC026356.1 knockdown repression significantly attenuated sphere formation of lung cancer cells (Figure 6C, 6D). Finally, we also showed that a AC026356.1 ceRNA regulated network in the progression of LUAD (Figure 6E, 6F). These data suggested that AC026356.1 modulate the stemness of LUAD cells.

### Correlation of AC026356.1 expression with LUAD immune cell infiltrates

**Figure 7:**
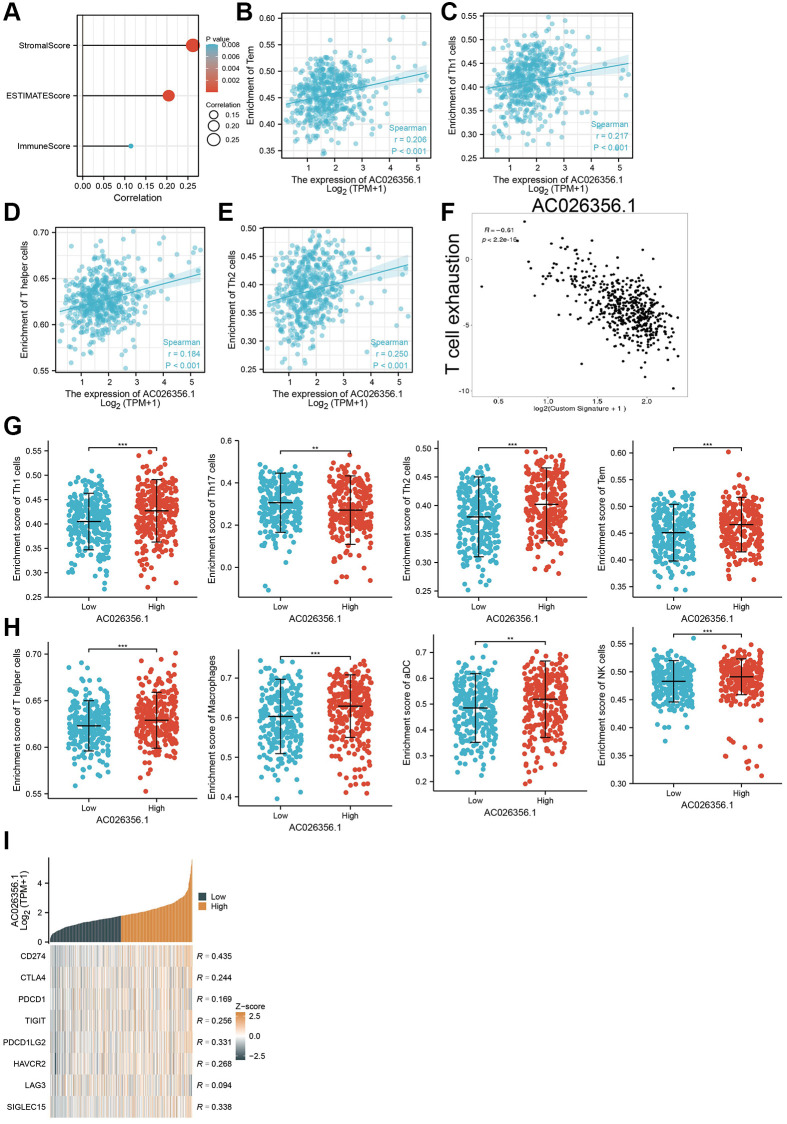
**Correlation between AC026356.1 expression and immune infiltrates.** (**A**) AC026356.1 expression was positively correlated with LUAD immune scores, stromal scores, and ESTIMATE scores. (**B**–**E**) Correlation between AC026356.1 expression and diverse immune infiltrates in LUAD. (**F**) Correlation between AC026356.1 expression and T cell exhaustion (**G**, **H**) The abundance of immune infiltrates of diverse immune cells based on the AC026356.1 high or low expression group. (**I**) Correlation between AC026356.1 expression and diverse immune checkpoint gene in LUAD. ^**^p < 0.01 and ^***^p < 0.001.

The dynamic changes in the immune microenvironment play a core role in tumor progression [15]. We will further explore the correlations between AC026356.1 expression and the TME of LUAD. First, we showed that AC026356.1 expression was positively related to the LUAD immune scores, stromal scores, and ESTIMATE scores (Figure 7A). High expression of AC026356.1 was positively related to the infiltration levels of Th1 and Tem cells in LUAD (Figure 7B–7E). T-cell exhaustion is considered to be an important factor in the malignant progression of tumors [16]. More importantly, we uncover that AC026356.1 was negatively correlated with T cell exhaustion (Figure 7F). Finally, we found that in the AC026356.1 -high expression group, the infiltration levels of Macrophage, Th1 cells, Th17, Th2, Tem, T helper, macrophage, ADC, and NK cell were significantly increased than AC026356.1-low expression group (Figure 7G, 7H). We also confirmed that AC026356.1 expression was significantly positively linked to CD274, CTLA4, PDCD1, TIGIT, PDCD1LG2, HAVCR2, and SIGLEC15 in LUAD (Figure 7I).

## DISCUSSION

In this finding, we uncover that AC026356.1 expression was decreased in LUAD, compared to normal lung tissues. Meanwhile, low AC026356.1 expression is associated with the cancer stage and overall event in LUAD. Prognosis analysis indicated that LUAD patients with high AC026356.1 levels were related to adverse OS and DSS in LUAD. Our results demonstrated that AC026356.1 can be used as a prognostic biomarker for LUAD.

In this study, expression level and phenotypic analyses (including proliferation and sphere formation assays revealed that AC026356.1 formed a positive loop to regulate the stemness of lung cancer. Here, we unveiled METTL14/IGF2BP2-mediated m6A modification of AC026356.1, which lead to its high expression in lung cancer. Mechanistic disruption of the METTL14/IGF2BP2/AC026356.1 loop provided experimental evidence of how the positive loop was formed and exerted stemness upon m6A modification. In our study, we showed that AC026356.1 is upregulated in LUAD cells more than in the bronchial epithelial cell line. *In vitro* cell experiments indicated that depletion of AC026356.1 significantly inhibited LUAD cell growth and migration.

Recent studies have reported that the three m6A modifiers investigated in this study promote the propagation of various cancer cells including lung cancer cells. METTL14 is highly expressed in distinct tumor types and boosts stemness by enhancing SOX2 mRNA stability upon m6A modification [17]. IGF2BP2 interacts with and stabilizes FEN1 mRNA from decay in liver cancer [18]. Our data revealed that METTL14/IGF2BP2-mediated m6A modification of AC026356.1 RNA rescued them from decay. The existence of cancer stemness is the main cause of drug resistance, and distal metastasis- and CSC-targeted therapies hold promise in the clinical treatment of cancers. AC026356.1 is also essential for stemness-related malignancy in lung cancer. *In vitro* experiments indicated that AC026356.1 activation leads to resistance of NSCLC cells to erlotinib treatment.

The lung cancer microenvironment depends on the complex interactions between tumor cells and diverse immune cells. TME is thought to be a key factor contributing to tumor progression and high mortality in patients [19, 20]. By performing the correlation analysis, we showed that AC026356.1 expression is associated with immune scores, stromal scores, and ESTIMATE scores. High expression of AC026356.1 was positively related to the infiltration levels of Th1 and T cell exhaustion.

The present study provides insights to improve CSC-targeted therapy in cancer. Combination therapy of chemo drugs or targeting drugs with inhibitors or nucleic acid drugs targeting the IGF2BP2/AC026356.1 loop *in vivo* may further help in identifying novel therapeutic sensitizers for multiple regimens of cancer treatment.
